# Schisandrol B protects against lithocholic acid-induced cholestatic liver injury in mice through mitochondrial biogenesis

**DOI:** 10.1186/s13020-026-01342-y

**Published:** 2026-04-14

**Authors:** Xiao Yang, Hangfei Liang, Xuan Li, Jianing Tian, Shicheng Fan, Min Huang, Jianbo Wan, Zhong Zuo, Haibiao Guo, Huichang Bi

**Affiliations:** 1 NMPA Key Laboratory for Research and Evaluation of Drug Metabolism & Guangdong Provincial Key Laboratory of New Drug Screening & Guangdong-Hongkong-Macao Joint Laboratory for New Drug Screening, School of Pharmaceutical Sciences, Southern Medical University, 1023# Shatai South Road, Baiyun District, Guangzhou, 510515 People’s Republic of China; 2 Guangdong Provincial Key Laboratory of New Drug Design and Evaluation, School of Pharmaceutical Sciences, Sun Yat-Sen University, Guangzhou, 510006 China; 3 State Key Laboratory of Mechanism and Quality of Chinese Medicine, Institute of Chinese Medical Sciences, University of Macau, Macao, China; 4 School of Pharmacy, Faculty of Medicine, The Chinese University of Hong Kong, Hong Kong, Special Administrative Region China; 5 Hutchison Whampoa Guangzhou Baiyunshan Chinese Medicine Co., Ltd., Guangzhou, 510515 Guangdong China

**Keywords:** Schisandrol B, Cholestatic liver injury, Mitochondrial biogenesis, Mitochondrial dynamics

## Abstract

**Background:**

Mitochondrial biogenesis plays a vital role in various types of hepatocyte injury. Schisandrol B (SolB), a bioactive lignan isolated from *Schisandra sphenanthera*, exerts a significant hepatoprotective effect against lithocholic acid (LCA)-induced cholestatic liver injury. Whether mitochondrial biogenesis is involved in the anti-cholestasis effect of SolB remains unknown.

**Methods:**

A mouse model of cholestatic liver injury was induced by intraperitoneal injection of LCA. SolB was administered orally twice a day. Serum alanine aminotransferase (ALT), aspartate aminotransferase (AST), alkaline phosphatase (ALP), total bile acids (TBA) and total bilirubin (TBILI), as well as hepatic superoxide dismutase (SOD) activity were measured. Liver pathology was evaluated by toxylin and eosin (H&E) staining. Mitochondrial morphology was examined using electron microscopy. Furthermore, the expression of mitochondrial biogenesis-related genes or proteins was analyzed by RT-qPCR or Western blot.

**Results:**

We confirmed that SolB pretreatment (200 mg/kg/d) alleviated LCA-induced liver injury as evidenced by histological and biochemical analyses. SolB alleviated LCA-induced mitochondrial dysfunction in mice, as evidenced by increased mitochondrial DNA (mtDNA) content, superoxide dismutase (SOD) levels, and peroxisome proliferator-activated receptor γ co-activator 1α (PGC-1α) and mitochondrially encoded cytochrome c oxidase subunit 1 (MTCO1) expression, together with decreased fibroblast growth factor 21 (*Fgf21*) and growth differentiation factor 15 (*Gdf15*) gene levels. Transmission electron microscope analysis showed that LCA elicited small, fragmented mitochondria, which were not reversed after SolB pretreatment. However, western blot analysis showed that the expression of mitochondrial dynamics-related proteins, such as dynamin-related protein1 (DRP1), optic atrophy 1 (OPA1), mitofusin 1 (MFN1), and MFN2, was significantly decreased after LCA treatment. Pretreatment with SolB could significantly upregulate DRP1, mitochondrial fission factor (MFF), and fission1 (FIS1) which are crucial to regulate mitochondrial fission. It is worth noting that the protective effect of SolB against LCA-induced liver injury was independent of parkin RBR E3 ubiquitin-protein ligase (PARKIN)-mediated mitophagy as evidenced by decreased PARKIN and microtubule-associated protein light chain 3 (LC3)-II.

**Conclusion:**

In summary, this study demonstrated that SolB improved mitochondrial function but had no effect on LCA-induced mitochondrial fragmentation, which provides new insights into better understanding hepatoprotective mechanism of SolB against cholestatic liver injury.

**Supplementary Information:**

The online version contains supplementary material available at 10.1186/s13020-026-01342-y.

## Introduction

Cholestatic liver injury is caused by dysfunction of bile acid metabolism resulting in intracellular accumulation of bile acids, bilirubin and cholesterol [1, 2]. Cholestasis can further develop into liver fibrosis and cirrhosis, end-stage liver injury and even death if treatment is not performed timely [3, 4]. It has been identified that mitochondrial dysfunction plays a critical role in the progression of cholestatic liver injury. The toxic bile acids induce mitochondrion-mediated apoptosis characterized by DNA fragmentation, chromatin condensation, cellular shrinkage and cell membrane blebbing [5, 6]. It has been reported that chronic cholestatic diseases may arise from apoptotic and necrotic cell death involving mitochondria [7, 8]. Moreover, the mitochondrial-mediated pathway was involved in hepatocyte apoptosis in α-naphthylisothiocyanate (ANIT)-induced cholestasis [9].

Mitochondria, also called the “powerhouse”, are highly dynamic organelles in cells. Mitochondrial dynamics are regulated by two sets of opposing processes: mitochondrial fusion and fission, and mitochondrial biogenesis and mitophagy [10]. These processes play an active role in protecting mitochondrial morphology and function [11–13]. Mitochondrial fission and fusion are mediated by the dynamin-related family of large GTPases. In mammals, fusion process is coordinated by optic atrophy 1 (OPA1), mitofusin 1 (MFN1) and MFN2 [13]. Dynamin-related protein 1 (DRP1) is responsible for mitochondrial fission [12]. When the balance between fission and fusion is disrupted, damaged mitochondria are selectively degraded through the mechanism of the phosphatase and tensin homolog (PTEN)-induced kinase 1(PINK1)/Parkin RBR E3 ubiquitin-protein ligase (PARKIN)-mediated mitophagy pathway [14]. Moreover, mitochondrial function factors (such as PGC-1α, TFAM, MTCO1, FGF21, GDF15) play important roles in reducing mitochondrial damage. Hepatocytes have a large number of mitochondria, indicating a key role of mitochondrial dynamics in hepatocyte injury and recovery [15]. Acetaminophen affects mitochondrial morphology by stimulating DRP1-mediated mitochondrial fission in primary hepatocytes and mice [16]. DRP1 inactivation can promote hyperfused megamitochondria formation in hepatoma VL-17A cells cultured with ethanol [17]. Glycochenodeoxycholate (GCDC) is a toxic bile salt and induces mitochondrial fragmentation in primary hepatocytes. Moreover, inhibition of mitochondrial fission prevents cell death after GCDC exposure and diminishes the liver injury after bile duct ligation [18]. However, appropriate fission facilitates the removal of damaged mitochondria, balances energy supply, and enables cells to adapt to stress, thereby exerting a protective effect [19]. Accumulating evidence has revealed that DRP1-mediated mitochondrial fission is a prerequisite for mitophagy, which is critical for the degradation of damaged mitochondria and has been identified as a key factor in regulating mitochondrial function and maintaining cell viability [20].

Schisandrol B (SolB), one of the most important bioactive components isolated from *Schisandra sphenanthera*, exerts hepatoprotective effects against various types of liver injury induced by carbon tetrachloride, alcohol and acetaminophen [21]. In addition, the liver-protective effect of the traditional Chinese medicine Hugan Tablets, which are mainly composed of *Schisandra sphenanthera*, has also been reported and confirmed by relevant studies [22–24]. Previously, we reported that SolB exerts a hepatoprotective effect against lithocholic acid (LCA)-induced cholestatic liver injury through activation of the pregnane X receptor (PXR) pathway and reverses abnormal bile acids profiles and alteration of gut microbiome [3, 25, 26]. However, whether mitochondrial quality control and clearance are involved in the anti-cholestatic liver injury effect of SolB remains unclear. Therefore, this study aims to investigate the role of mitochondria in the hepatoprotection of SolB against LCA-induced cholestatic liver injury and explore its underlying mechanisms.

## Materials and methods

### Reagents

LCA (purity 98%) was obtained from Aladdin Company (Shanghai, China). Schisandrol B (purity 98%) was purchased from Shanghai Winherb Medical Science and Technology Development Co. Ltd. (Shanghai, China). Antibodies used in the study included DRP1 (BD Biosciences, 611112); OPA1 (Abcam, ab157457); MFN1 (Abcam, ab57602); MFN2 (Santa Cruz Biotechnology, 515647); MFF (Proteintech, 17090-1-AP); FIS1 (Proteintech, I0956-1-AP); phospho-DRP1 (Ser637) (Cell Signaling Technology, 6319); Phospho-DRP1 (Ser616) (Cell Signaling Technology, 4494); PINK1 (Abcam, ab23707); PARKIN (Abcam, ab77924); RIP1 (Cell Signaling Technology, 3493); RIP (Phospho Ser166) (Immunoway, YP1467); RIP3 (Cell Signaling Technology, 95702); Phospho-RIP3 (Thr231/Ser232) (Cell Signaling Technology, 91702); MLKL (Cell Signaling Technology, 37705S); Phospho-MLKL (Ser345) (Cell Signaling Technology, 37333); p62 (Cell Signaling Technology, 5114); LC3 (Cell Signaling Technology, 12741); FUNDC1 (Abcepta, AP17377a); NRF2 (ABclonal, A1244); HO-1(ABclonal, A1346); NQO1 (Immunoway, YM8039); GCLC (Immunoway, YM8420); GCLM (Immunoway, YM8359); GAPDH (Cell Signaling Technology, 2118). Peroxidase-conjugated anti-rabbit and anti-mouse immunoglobulin G (IgG) were purchased from Cell Signaling Technology (Danvers, MA, USA).

### Animals and treatment

Male C57BL/6J mice (6–8 weeks old) were obtained from Guangdong Medical Laboratory Animal Center (Guangzhou, China). All animals received humane care in Laboratory Animal Service Center of Sun Yat-sen University (Guangzhou, China). All procedures were approved by the Animal Ethics and Welfare Committee of Sun Yat-sen University. The mice were randomly grouped into 4 groups: the control group (n = 6), the SolB (100 mg/kg, bid, n = 6) group, the LCA (125 mg/kg, bid, n = 10) group, SolB + LCA group (n = 8). According to our previous descriptions, SolB (100 mg/kg) was dissolved in 0.5% carboxymethylcellulose sodium and LCA (125 mg/kg) was dissolved in corn oil [3, 34]. Mice in the SolB group and LCA + SolB group were given SolB by gavage twice daily for 7 days, while mice in the control and LCA groups received the same volume of 0.5% carboxymethylcellulose sodium by gavage. LCA was injected intraperitoneally from the fourth day for 4 days, mice in the control and SolB groups received the same volume of corn oil. Mice were sacrificed at 12 h after LCA injection, but 6 mice in the LCA group died before this time point.

Serum and liver tissues were harvested and stored at -80°C for further analysis. Paraffin-embedded sections of the liver were stained with Hematoxylin and Eosin (H&E) and RIP3 antibody. The levels of serum alanine aminotransferase (ALT), aspartate aminotransferase (AST), alkaline phosphatase (ALP), total bile acids (TBA) and total bilirubin (TBILI) were measured as described previously [3, 34, 73]. Total SOD levels in the liver were measured using a SOD assay kit (A001-3-2, Nanjing Jiancheng Bioengineering Institute, Nanjing, China).

### Western blot analysis

Western blots were performed according to our published reports with slight modifications [74, 75]. In brief, protein extracts from mice liver tissue were prepared using RIPA lysis buffer (R0127, Biocolors, Shanghai, China) referring to the manufacturer’s instructions. Protein concentrations were measured using the Pierce BCA Protein Assay Kit (23225, Thermo Fisher Scientific, Waltham, USA). Protein was separated by SDS polyacrylamide gel electrophoresis before being transferred onto membranes (ISEQ00010, Millipore, Bedford, USA). Membranes were blocked for 1 h at room temperature and then probed with the indicated primary antibody, followed by incubation with secondary anti-rabbit or anti-mouse antibodies at room temperature for 1 h. Furthermore, immunodetection was performed using an electrochemiluminescence (ECL) kit (WBKLS0500, Millipore, Bedford, USA). Protein band intensities were analyzed with the Image J software (National Institutes of Health, Bethesda, MD, USA).

### Real-time quantitative PCR analysis (RT-qPCR)

According to our previously described method [76], total RNA was extracted from mice liver tissue samples using TRizol reagent (15596-018, Invitrogen, New York, USA) and reverse-transcribed into complementary DNA by using the Evo M-MLV RT Premix Kit (AG11706, Accurate Biotechnology, Changsha, China). RT-qPCR analysis was performed on an Applied Biosystems 7500 real-time PCR system (Applied Biosystems, Foster City, USA) using the SYBR Green Pro Taq HS qPCR kit (AG11701, Accurate Biotechnology, Changsha, China). Primers are listed in Table 1.

**Table 1:**
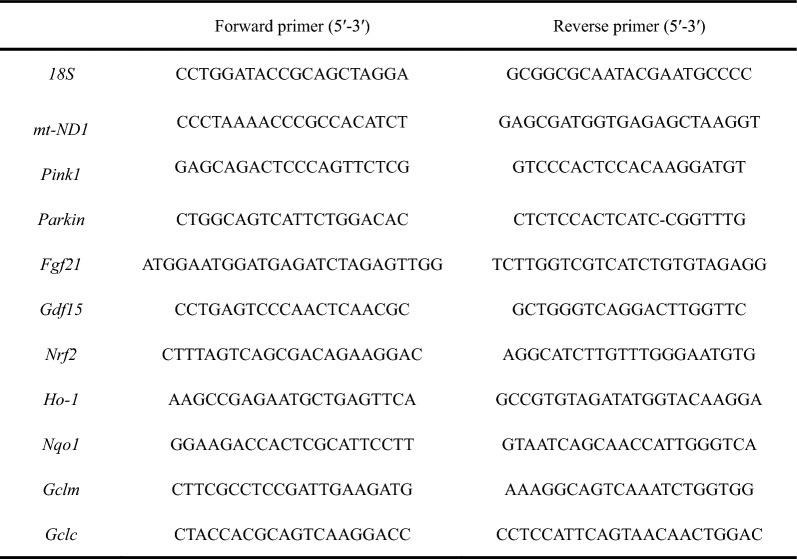
Sequences of primers for qPCR

### Electron microscopy (EM) analysis

After designated treatments, mice liver samples were fixed with 2.5% glutaraldehyde in 0.1 mol/L phosphate buffer (pH 7.4), followed by 1% OsO_4_. After dehydration, thin sections were stained with uranyl acetate and lead citrate and then examined with a JEM 1011CX electron microscope (JEOL) as described previously [12].

### Mitochondrial DNA content detection

The mitochondrial DNA (mtDNA) copy number was assessed by determining the ratio of mtDNA-encoded *ND1* to nuclear-encoded *18S* using real-time PCR. DNA was extracted using the DNeasy Blood and Tissue Kit (Qiagen) according to the manufacturer’s instructions. The relative copy numbers were quantified on an Applied Biosystems 7500 real-time PCR system (Applied Biosystems, Foster City, USA) using the SYBR Green Pro Taq HS qPCR kit (AG11701, Accurate Biotechnology, Changsha, China). 

### Statistical analysis

All experimental data were expressed as the mean ± standard deviation (SD). Statistical analysis was performed by one-way analysis of variance (ANOVA). *P* values < 0.05 were considered statistically significant.

## Results

### SolB attenuates LCA-induced cholestatic liver injury in mice

**Fig. 1:**
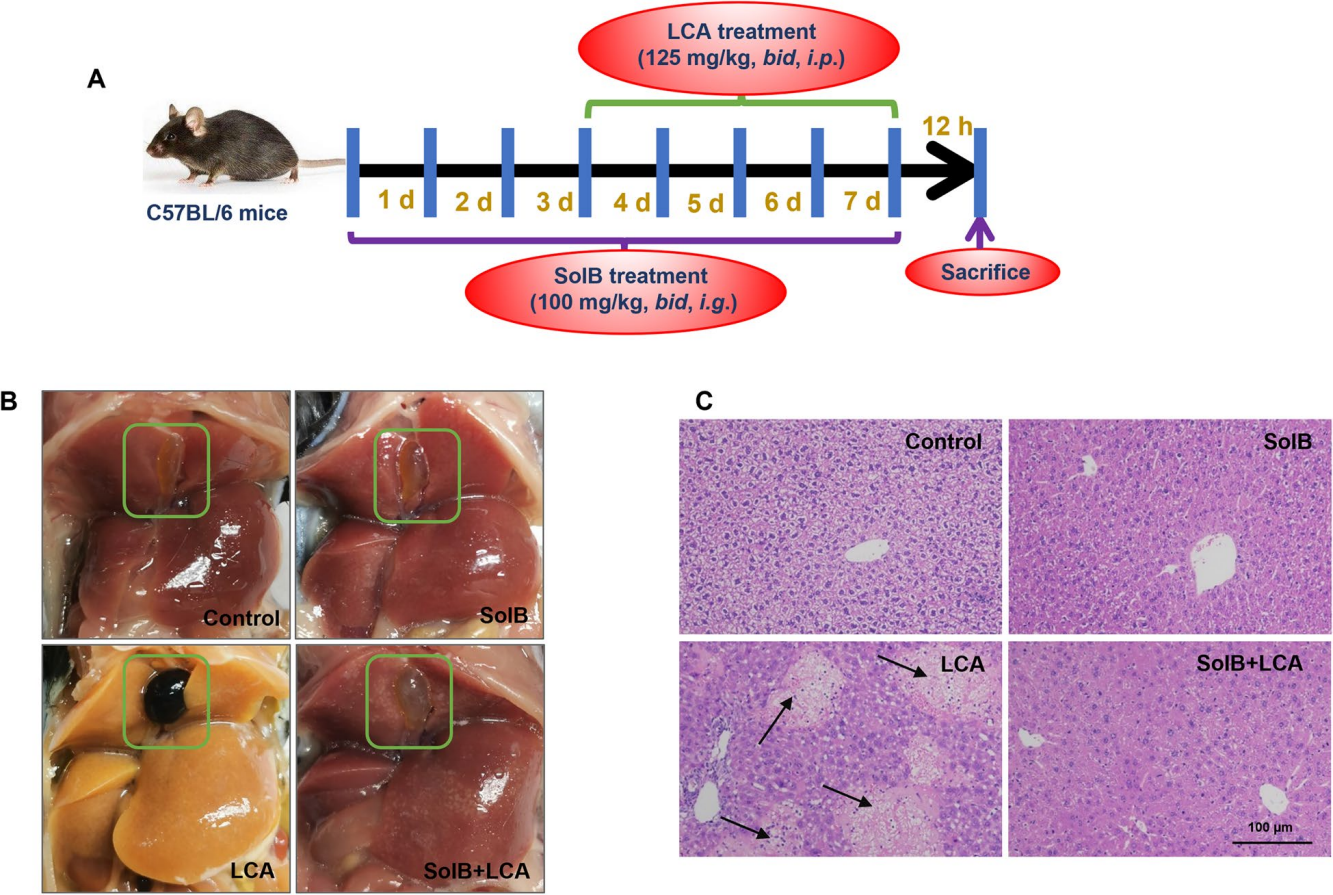
Effect of SolB on the liver in LCA-induced cholestasis mice. Male C57BL/6 mice were treated with SolB (200 mg/kg/d) by gavage for 7 days, and LCA (250 mg/kg/d) was injected intraperitoneally from the fourth day for 4 days. Mice were sacrificed at 12 h after LCA injection. **A** Schematic representation illustrates the experimental design in the mouse model. **B** Representative images of livers in situ. Gall bladders were boxed. **C** Representative images of H&E-stained liver sections. Arrows denote injury zones. Scale bar: 100 μm

**Fig. 2:**
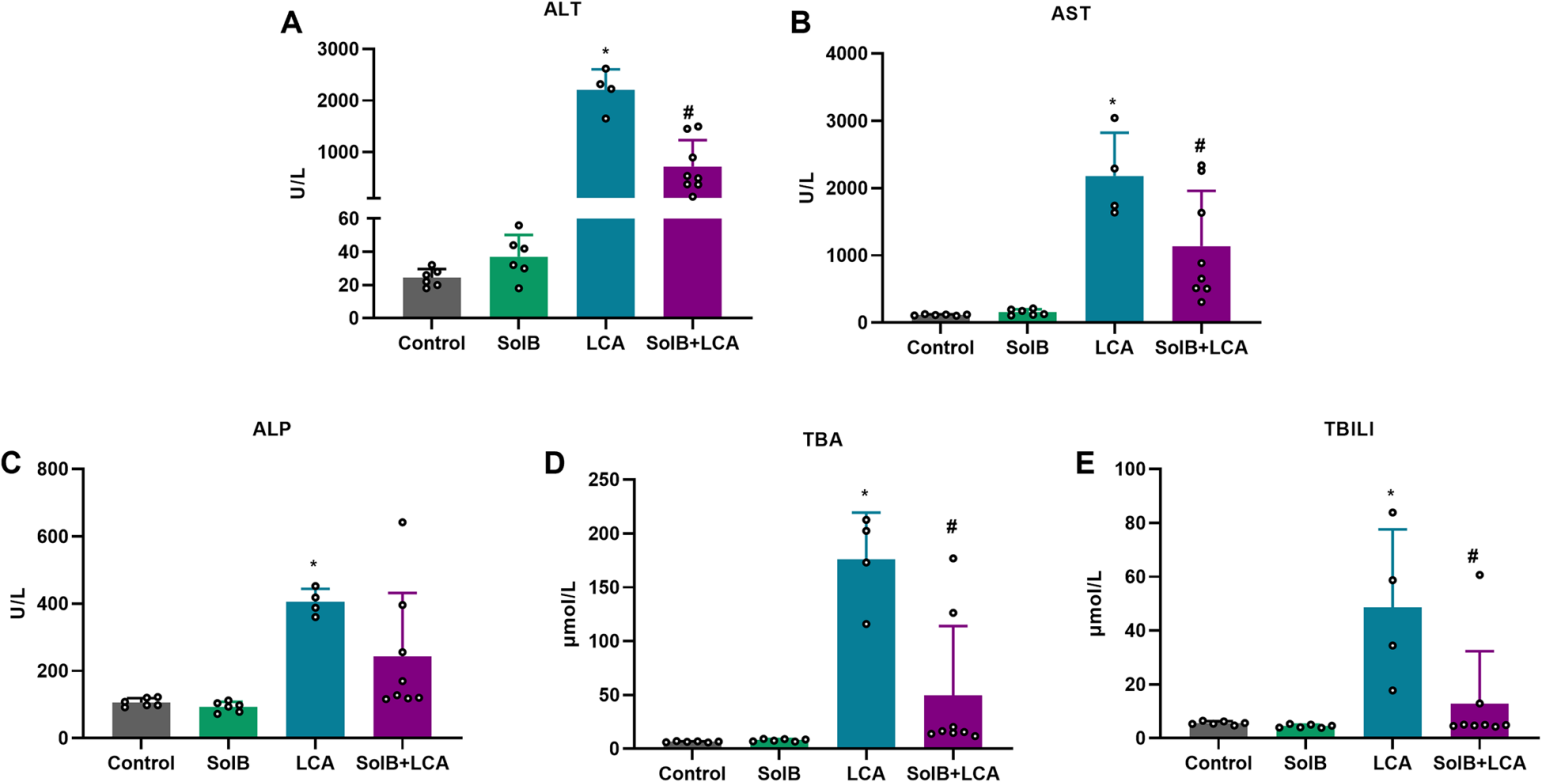
Effect of SolB on the serum biochemical markers induced by LCA. **A**–**C** Serum ALT, AST and ALP levels. **D**, **E** Serum TBA and TBILI levels. Data are the mean ± SD (n = 4–8). ^*^P < 0.05 versus the control group, ^#^*P* < 0.05 versus the LCA group

To confirm the effect of SolB on LCA-induced cholestatic liver injury, male mice were treated with LCA at 250 mg/kg/d in the absence or presence of 200 mg/kg/d SolB (Fig. 1A). Morphological results showed gallbladder enlargement, darkened bile, striking hepatic necrosis, extensive cytoplasmic vacuolization and infiltrating neutrophils after LCA dosing, but SolB pretreatment obviously reversed these morphological changes as illustrated by the H&E staining of liver tissues (Fig. 1B, C). Serum ALT, AST, and ALP levels in the LCA group were significantly elevated 90.5-, 18.9-, and 3.8-fold relative to the controls, respectively; these elevations were reduced to 32.5%, 52.2% and 60.1% after SolB pretreatment (Fig. 2A–C). LCA treatment significantly increased serum TBA and TBILI levels to 27.3- and 8.6-fold higher than that of the control mice, which was significantly reduced to 28.1% and 26.2% after SolB treatment (Fig. 2D, E). These results are in general consistent with our previous study, which showed that SolB exerts the hepatoprotection against LCA-induced cholestatic liver injury in mice [2, 3, 25].

**Fig. 3:**
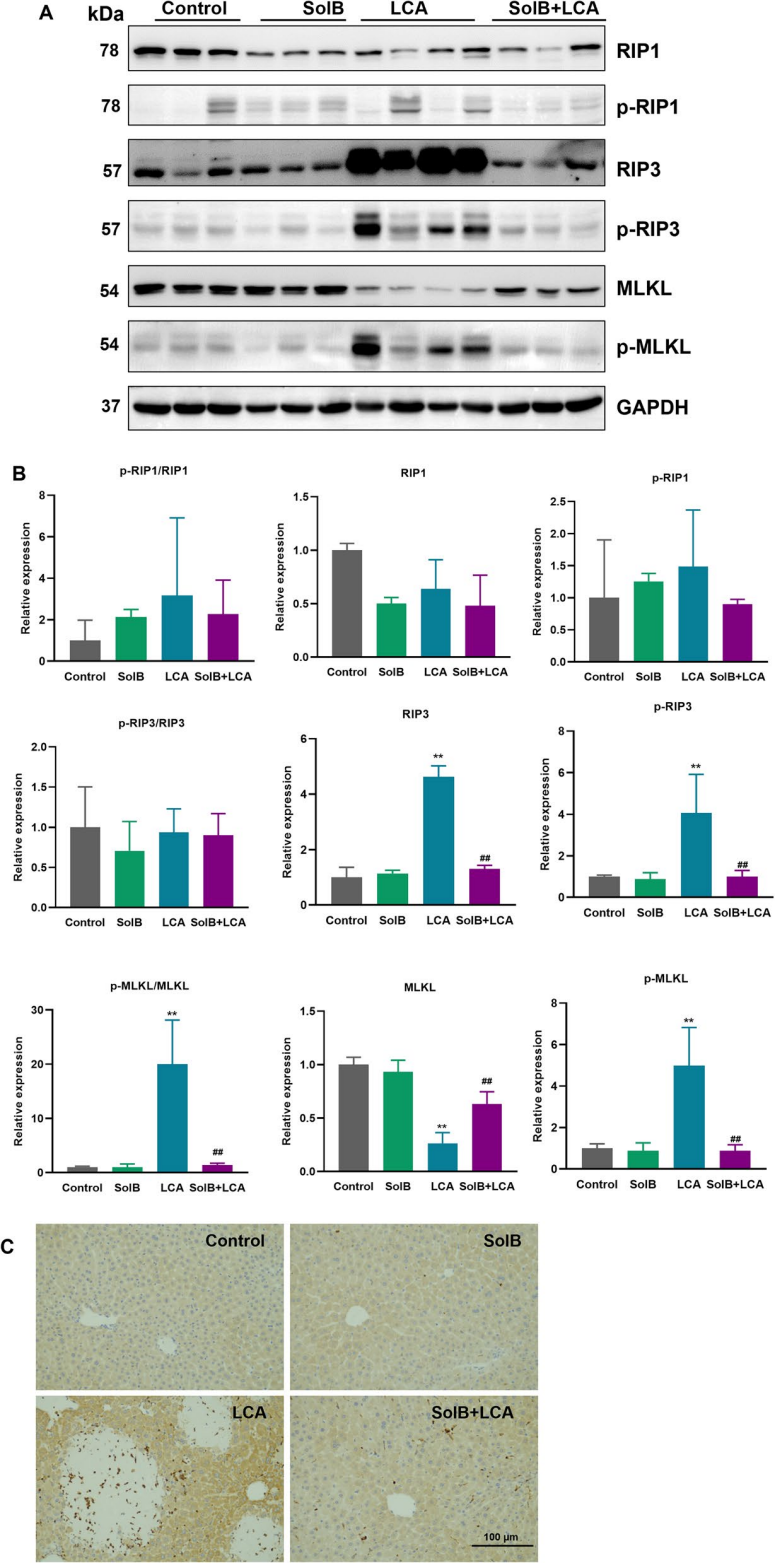
Effect of SolB on the RIP1/RIP3/MLKL signaling pathway in mice. **A** The expression levels of RIP1, p-RIP1, RIP3, p-RIP3, MLKL, and p-MLKL in livers were determined by Western blot analysis. **B** Densitometry analysis of (A). The data are presented as mean ± SD (n = 3–4), ^*^P < 0.05, ^**^P < 0.01 versus the control group; ^#^*P* < 0.05, ^##^*P* < 0.01 versus the LCA group. **C** Paraffin-embedded liver tissues were subjected to immunohistochemistry for RIP3. Scale bar: 100 μm

It has been reported that activation of necroptosis is emerging as a critical pathogenesis in human and experimental cholestasis [27–29]. To evaluate the effect of SolB on LCA-induced necroptosis, we determined levels of receptor-interacting protein1 (RIP1), receptor-interacting protein3 (RIP3) and mixed-lineage kinase domain-like protein (MLKL), which regulate necroptosis. The results showed that the protein levels of hepatic RIP1 and MLKL were significantly decreased in the LCA-treated mouse livers compared to those of the control group (decreased to 63.9% and 26.4% of control levels, respectively). The protein level of RIP3 after LCA treatment was 4.6-fold higher than that of the control group (Fig. 3A, B). Similarly, immunohistochemical analysis also showed marked induction of RIP3 in necrotic areas of LCA-treated mouse livers, which was not observed in the control group (Fig. 3C). Compared with the control group, the ratios of phospho-RIP1/RIP1 and phospho-RIP3/RIP3 remained unchanged in the LCA-treated mice, whereas the phospho-MLKL/MLKL ratio was markedly elevated; this increase was abolished by SolB pretreatment (Fig. 3A, B). SolB pretreatment obviously reversed RIP3 and MLKL levels changed by LCA treatment, but no difference in RIP1 levels. These data suggest that the levels of hepatic RIP3 and MLKL were differentially regulated by LCA, and the hepatoprotective effects of SolB against LCA-induced cholestatic liver injury may be partially through the RIP3-MLKL pathway.

### SolB alleviates the mitochondrial dysfunction induced by LCA in mice

**Fig. 4:**
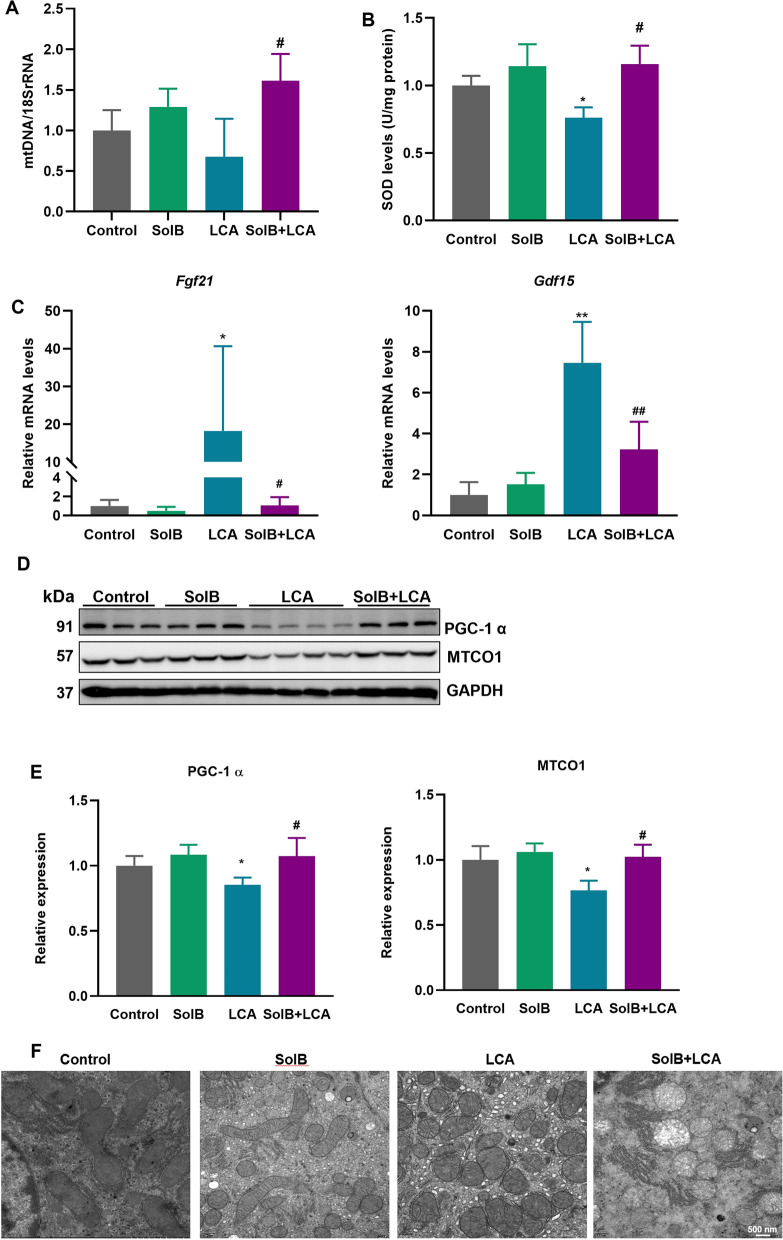
SolB alleviates the mitochondrial dysfunction induced by LCA in mice. **A** Relative mtDNA copy number (NADH dehydrogenase subunit 1, ND1) in mouse livers, as determined by qPCR.** B** Quantification of hepatic SOD levels. **C** The hepatic mRNA levels of *Fgf21* and *Gdf15*. **D**, **E** Western blot analysis and densitometric analysis of hepatic PGC1α , MTCO1 (n = 3–4). **F** Representative electron microscope (EM) photographs of hepatocytes. ^*^P < 0.05, ^**^P < 0.01 versus the control group; ^#^*P* < 0.05, ^##^*P* < 0.01 versus the LCA group

To evaluate the mitochondrial functions, mtDNA, superoxide dismutase (SOD) levels and expression of mitochondrially encoded cytochrome c oxidase subunit 1 (MTCO1), peroxisome proliferator-activated receptor-γ co-activator 1α (PGC-1α), mitostress genes fibroblast growth factor 21 (*Fgf21*) and growth differentiation factor 15 (*Gdf15*) were determined. As shown in Fig. 4A, SolB pretreatment also elevated mtDNA levels in LCA-treated mice. The levels of mitostress genes *Fgf21* and *Gdf15* were elevated in the LCA group (18.2-fold and 7.5-fold versus the control group), but this elevation was significantly reduced by SolB pretreatment (Fig. 4C). PGC-1α regulates the mitochondrial biogenesis. The expression of PGC-1α and MTCO1 was downregulated by 14.7% and 23.5% after LCA treatment compared to that of the control group, which was obviously reversed by SolB pretreatment (Fig. 4D, E). SOD levels were significantly decreased in the LCA group (76.0% of control) but were substantially increased by SolB pretreatment (1.5-fold versus the LCA group, Fig. 4B). In addition to SOD, the expression levels of other oxidative stress-related genes such as nuclear factor erythroid 2-related factor 2 (NRF2), NAD(P)H quinone oxidoreductase 1 (NQO1), heme Oxygenase 1 (HO-1), glutamate-cysteine ligase modifier subunit (GCLM), and glutamate-cysteine ligase catalytic subunit (GCLC) were also determined. The levels of *Nqo1*, *Ho-1*, *Gclm,* and *Gclc* were increased in the LCA group, which was decreased after SolB pretreatment (Supplementary Fig. 1A). Protein analysis showed that the expression levels of NRF2, HO-1, and NQO1 were significantly upregulated in the LCA group. Pretreatment with SolB reduced the expression of HO-1 and NQO1. Additionally, NQO1 expression was higher in the SolB alone group than in the control group (Supplementary Fig. 1B–C).

Furthermore, results from electron microscopy(EM) showed the presence of mitochondrial swelling and fragmented, shorter mitochondria in the mouse livers with exposure to LCA. However, these alterations were not changed after SolB pretreatment (Fig. 4F). Taken together, the above results indicate that SolB could alleviate the mitochondrial dysfunction induced by LCA, but had no effect on mitochondrial fragmentation in hepatocytes from LCA‑treated mice.

### Effect of SolB on the expression of proteins related to mitophagy

**Fig. 5:**
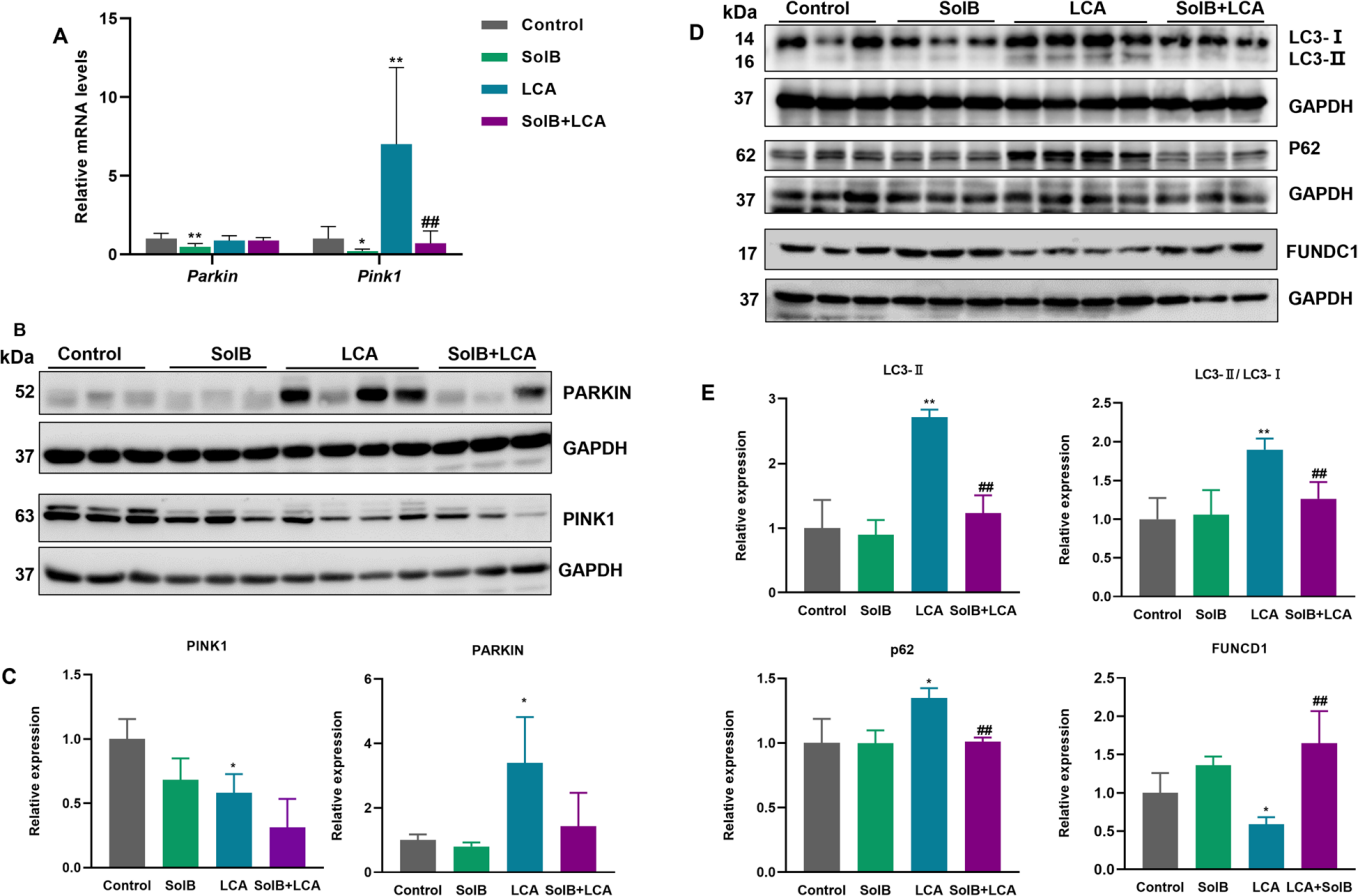
Effect of SolB on the proteins related to mitophagy. **A** The mRNA expression of *Pink1*, *Parkin* was analyzed by qPCR, and values are the mean ± SD (n = 4–6).** B**, **C** Western blot analysis and densitometric analysis of PINK1, PARKIN in livers (n = 3–4). **D**, **E** Western blot analysis and densitometric analysis of LC3, p62, and FUNDC1 in livers (n = 3–4). ^*^P < 0.05,^**^P < 0.01 versus the control group; ^#^*P* < 0.05, ^##^*P* < 0.01 versus the LCA group

To address whether mitophagy pathway is involved in the protective effect of SolB against LCA-induced liver injury, we further determined the critical proteins of PINK1/PARKIN-mediated mitophagy in the mouse livers. As shown in Fig. 5A, after LCA treatment, *Pink1* expression was significantly increased to 7.0-fold of the control group, no marked changes were observed in *Parkin* expression. SolB alone significantly decreased the *Pink1* and *Parkin* expression (to 21.4% and 49.1%, respectively). However, SolB pretreatment only significantly downregulated *Pink1* level (to 10%) and had no effect on *Parkin* compared to LCA-treated mice. Furthermore, the protein levels of PINK1 and PARKIN were determined (Fig. 5B, C). Consistent with the mRNA levels, PINK1 and PARKIN were downregulated to 68.2% and 80.1% in SolB-treated mouse livers compared with those of the control mouse livers. PINK1 expression was decreased to 58.1% after LCA treatment, whereas significant increase in PARKIN expression (3.4-fold). Furthermore, increased PARKIN level in the LCA group was diminished to 12.4% of the control after SolB pretreatment, while PINK1 levels were reduced to 53.8%.

The microtubule-associated protein light chain 3 (LC3) and p62/SQSTM1 are well-known markers of autophagy flux [30]. As shown in Fig. 5D, E, LCA treatment markedly increased LC3-II level (2.7-fold) and the ratio of LC3-II/LC3-I (1.9-fold) compared with those of the control group. The ratio of LC3-II/LC3-I was decreased to 66.7% after SolB pretreatment. FUN14 domain-containing protein 1 (FUNDC1) is one of the reported mitophagy receptor protein, which plays an important role in mitophagy [31]. Compared to the control group, expression of FUNDC1 protein was decreased to 59.2% in the LCA group, which was upregulated after SolB pretreatment (2.7-fold). Interestingly, the level of p62, a selective autophagic adaptor, was also increased by LCA (1.3-fold). Increased levels of LC3-II and p62 by LCA treatment were attenuated by SolB pretreatment. These results suggest that the protective effect of SolB against LCA-induced liver injury is likely independent of PARKIN-mediated mitophagy.

### Effect of SolB on expression of proteins related to mitochondrial dynamics

**Fig. 6:**
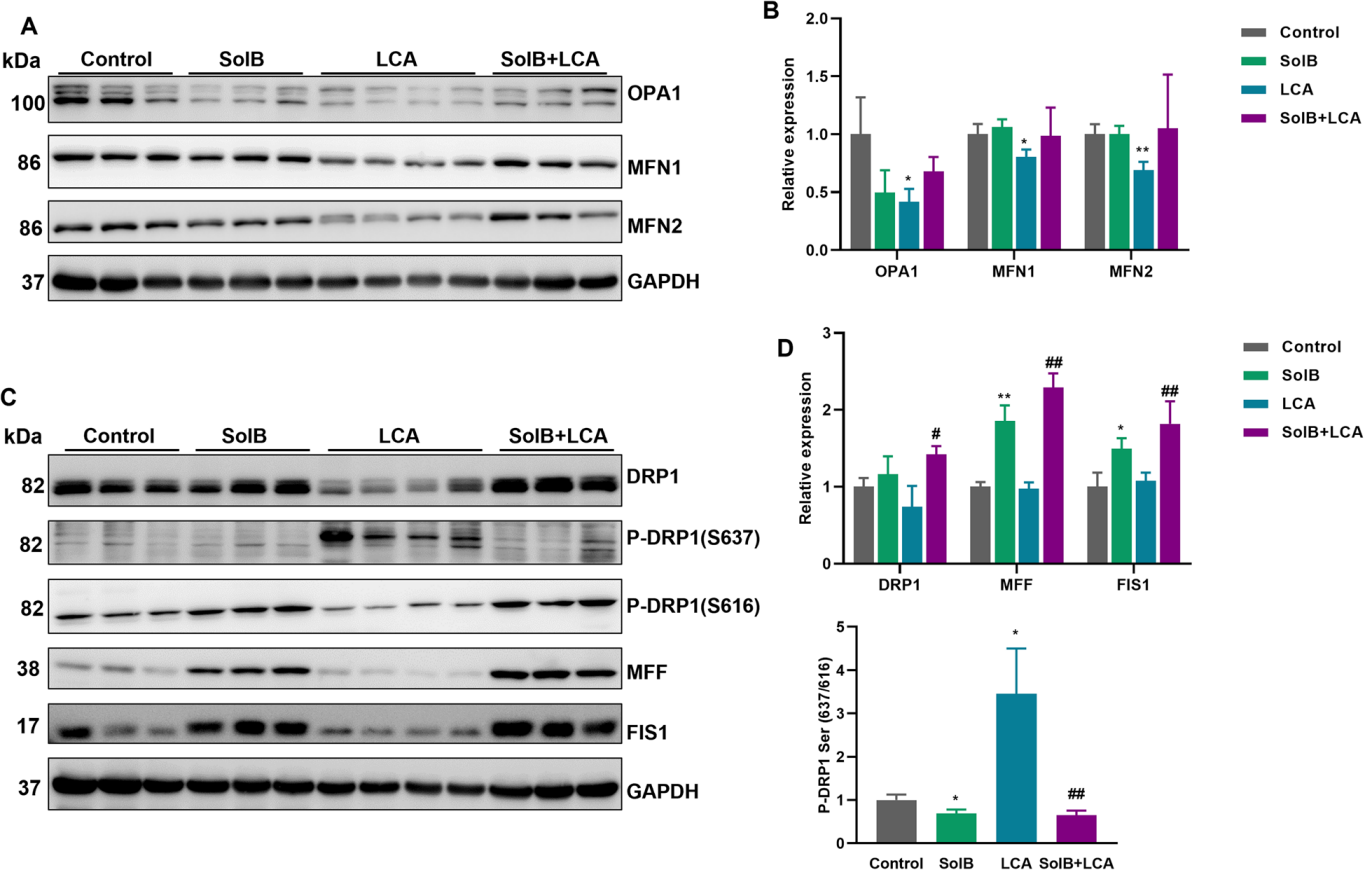
Effect of SolB on the proteins related to mitochondrial dynamics. **A**, **B** Western blot analysis and densitometric analysis of proteins related to mitochondrial fusion in livers. **C**, **D** Western blot analysis and densitometric analysis of proteins related to mitochondrial fission. The data are presented as mean ± SD (n = 3–4), ^*^P < 0.05, ^**^P < 0.01 *versu*s the control group; ^#^*P* < 0.05, ^##^*P* < 0.01 versus the LCA group

During mitophagy, the damaged mitochondria are separated from their healthy part by mitochondrial fission, which is a critical part of mitochondrial dynamics [32]. To further verify the possible effects of SolB on the mitochondrial morphology in LCA-induced cholestatic liver injury, we analyzed the mitochondrial dynamic-associated proteins involved in mitochondrial fission and fusion. Immunoblotting analyses showed that the levels of critical proteins involved in mitochondrial fusion such as OPA1, MFN1 and MFN2 were markedly decreased after LCA dosing (41.6%, 80.4% and 69.1% of the control group, respectively) (Fig. 6A, B). Although there was no significant difference, the fission protein DRP1 was also downregulated to 73.9% of the control group. It is known that in general, phosphorylation at Ser637 inhibits Drp1 activity while phosphorylation at Ser616 activates Drp1 [33]. The DRP1 Ser637/Ser616 phosphorylation ratio was increased to 3.5-fold in the LCA group, which indicates the DRP1 function is inhibited. MFF and FIS1 expression levels had no difference after LCA treatment (Fig. 6C, D).

Compared with those in the LCA group, SolB pretreatment significantly upregulated levels of DRP1, MFF and FIS1 (1.9-fold, 2.3-fold and 1.7-fold, respectively). The DRP1 Ser637/Ser616 phosphorylation ratio was significantly decreased to 18.8% (Fig. 6D). The fusion proteins OPA1, MFN1, and MFN2 were upregulated (1.6-, 1.2- and 1.5-fold, respectively) (Fig. 6B). In addition, we found that SolB treatment alone decreased levels of OPA1 (49.7%) and the DRP1 Ser637/Ser616 phosphorylation ratio (69.2%), whereas expression levels of FIS1 (1.5-fold) and MFF (1.9-fold) were increased. These results suggest that the downregulation of proteins involved in mitochondrial fusion may contribute to the mitochondria fragmentation induced by LCA.

## Discussion

Cholestatic liver injury can further develop into serious liver diseases and is a threat to human health. LCA, as one of the most toxic bile acids, is generated by bacterial 7α-dehydroxylation of chenodeoxycholic acid in the intestine [34]. Level of LCA are elevated in circulating blood of cholestatic patients, and hepatic parenchymal damage and disruption of bile flow induced by LCA are similar to those in humans with cholestatic liver disease [35]. Therefore, LCA-induced cholestatic animal models have been widely used to study intrahepatic cholestasis. In the present study, we found that liver damage induced by LCA was markedly alleviated by SolB pretreatment as evidenced by liver morphology and histological assessment, as well as biochemical analyses. All of the above data were in line with our previous results, suggesting that SolB has a significant hepatoprotective effect against LCA-induced intrahepatic cholestasis [2, 3]. The reduction in ALP was not statistically significant, which was due to individual differences in mice. Moreover, we found that SolB pretreatment could improve the function of mitochondria and reverse expression levels of proteins associated with mitochondrial biogenesis. Hence, mitochondria could be responsible for the mechanism of hepatoprotection of SolB against LCA-induced liver injury.

Necroptosis (also called programmed cell necrosis) is mediated by the RIP1/RIP3/MLKL pathway, and is emerging as a critical pathogenic mechanism in several liver diseases including cholestatic liver injury [28]. Necroptosis was activated in the liver of patients with primary biliary cholangitis (PBC) [27, 36]. Furthermore, it has been reported that necroptosis is triggered in the liver of mice subjected to bile duct ligation as evidenced by activation of RIP3 and MLKL simultaneously, RIP3 deficiency prevents necroinflammation induced by bile duct ligation in mice [27]. In the present study, SolB attenuated the upregulation of RIP3, phospho-RIP3 and phospho-MLKL. Interestingly, total RIP1 was down-regulated by either LCA or SolB alone, whereas the phospho-RIP1/RIP1 ratio increased after LCA exposure and was attenuated, though not significantly, by SolB pretreatment. This observation is consistent with previous reports that pharmacologic inhibition of RIP1 mitigates cholestatic injury, hepatic inflammation and biliary fibrosis in murine primary sclerosing cholangitis (PSC) [37]. We speculate that this subtle change reflects the stage of injury. In our model, LCA- driven liver damage has already progressed to the middle-late phase, when RIP1 phosphorylation is no longer markedly elevated. Collectively, our findings suggest that the hepatoprotective effect of SolB against LCA-induced liver injury is mediated, at least in part, by inhibition of the RIP3/MLKL-dependent signalling pathway. However, how SolB reverses the decrease in MLKL and the increase in RIP3 in LCA models remains to be elucidated.

In addition to being key operators in the regulation of cell death including necroptosis, mitochondria are also recognized as a primary target of toxic bile acids [38, 39]. Accumulating evidence suggests that mitochondrial dysfunction and oxidative stress play a significant role in the development of cholestatic liver disease [5, 40–42]. Various models of mitochondrial stress result in induction of the stress-responsive cytokines FGF21 and GDF15 [43]. In the current study, SolB attenuated the LCA-induced increase in *Fgf21* and *Gdf15* mRNA levels in the livers of mice challenged with LCA. Excessive ROS generation can result in mitochondrial dysfunction and cell death. Long-term cholestasis in the rats was related to the decrease in functions of liver mitochondria [44]. In mouse models of obstructive cholestasis, the livers exhibited lower expression of PGC-1α, which plays a crucial role in the induction of oxidative stress [45]. In addition, PGC-1α also upregulates the mitochondrial antioxidant pathways [46]. In this study, SolB restored the decrease in PGC-1α and SOD levels in the livers of mice challenged with LCA. Furthermore, the present study revealed that pretreatment with SolB restored the MTCO1 level that were reduced by LCA treatment. MTCO1 is one of the three mtDNA-encoded subunits of mitochondrial respiratory complex IV and considered as an indirect indicator of activity and quantity of mtDNA [47, 48]. These results indicate that the hepatoprotective effect of SolB against LCA-induced liver injury involves the inhibition of oxidative stress and the attenuation of mitochondrial dysfunction.

Previous studies have shown that LCA increases nuclear accumulation of NRF2 and induces multiple NRF2 target genes [49]. In this study, SolB administration attenuated the upregulation of NRF2 downstream target genes (HO-1 and NQO1) triggered by LCA. This aligns with reports that NRF2-driven HO-1 upregulation enhances bilirubin synthesis and accumulation, worsening liver injury in oleanolic acid (OA)- or α-naphthylisothiocyanate (ANIT)-induced cholestasis [50, 51]. However, this appears to contradict previous findings showing that sustained NRF2 activation is hepatoprotective against cholestasis-related liver injury [52, 53]. The induction of NRF2 downstream antioxidant genes has also been reported to effectively reduce oxidative stress-induced liver injury in the ANIT model [53]. We previously found that SolB increases the nuclear accumulation of NRF2 and increases hepatic expression of the NRF2 downstream proteins (NQO1) in APAP-treated mice [54]. In line with this, the current study also showed that SolB treatment upregulated NQO1 protein expression. It is therefore worthwhile to explore whether the effect of SolB on the NRF2 signaling pathway depends on the liver injury model or the extent of hepatic damage. Such model-dependent differences underscore the need for further mechanistic studies to clarify the dual roles of NRF2 activation in different contexts of cholestatic liver injury.

Mitochondria are network structures that are molded by the opposing processes of fission and fusion. Mitochondrial fission, as the division process within mitochondrial dynamics, is a key mechanism for maintaining mitochondrial health. Aberrant mitochondrial morphology has been implicated in many human diseases [55]. DRP1 possesses two critical phosphorylation sites Ser616 and Ser637. DRP1-S616 phosphorylation or DRP1-S637 dephosphorylation promotes DRP1 oligomerization and subsequently drives mitochondria constriction and cleavage [56, 57]. Inhibition of mitochondrial fission could, to some extent, alleviate certain types of liver injury. However, appropriate fission facilitates the removal of damaged mitochondria, balances energy supply, and enables cells to adapt to stress, thereby exerting a protective effect [19]. In this study, we found SolB improved mitochondrial function but did not alter LCA-induced mitochondrial fragmentation. Concurrently, SolB pretreatment reduced the LCA-induced elevation of the DRP1 Ser637/Ser616 phosphorylation ratio, implying that SolB may promote DRP1-mediated mitochondrial fission; however, the underlying mechanism remains to be elucidated. Our observation is similar to a recent study in which suppression of DRP1 activity/expression can induce neuronal cell death, while DRP1 overexpression has no effect [58].

Accumulating evidence has revealed that DRP1-mediated mitochondrial fission is a prerequisite for mitophagy, which is critical for the degradation of damaged mitochondria and has been identified as a key factor in regulating mitochondrial function and maintaining cell viability [20]. Given the critical role of mitophagy in cholestasis-induced liver injury, we hypothesized that SolB pretreatment could selectively remove damaged mitochondria via mitophagy in LCA-challenged mice. Two gene products mutated in familial parkinsonism, PINK1 and PARKIN, function together to degrade damaged mitochondria through mitophagy [59]. PINK1 accumulates on the surface of dysfunctional mitochondria where it simultaneously recruits and activates PARKIN [60, 61]. In the present study, we found that PARKIN was upregulated by LCA, but not PINK1, in mouse livers. Interestingly, the hepatic mRNA levels of *Pink1* were increased after LCA dosing, but not *Parkin,* in mouse livers. Although it remains to be studied in the future how transcription of *Pink1* and *Parkin* is differentially regulated by LCA, current data suggest that LCA regulates PARKIN at the post-translational level. It should be noted that PINK1 levels were already decreased in LCA mouse livers, but mRNA levels of *Pink1* were increased. PINK1 is cleaved by proteases in the mitochondrial matrix, and PINK1 interacts directly or indirectly with specific members of the endoplasmic reticulum (ER)-associated degradation machinery, which results in PINK1 degradation [62]. It remains to be studied whether LCA decreases PINK1 levels through proteases or the ER-associated degradation.

FUNDC1 is a mammalian mitophagy receptor that interacts with and recruits LC3 to mitochondria for mitophagy [63]. Our data showed that LCA induced the increase in ratio of LC3-II/LC3-I and the decrease in FUNDC1 levels, suggesting that the elevation in PARKIN is a consequence of mitophagy. Moreover, p62 expression was also upregulated, which differs from some previous reports [64–66]. p62 is a selective autophagy adaptor protein, accepting the ubiquitinated cargoes via its C-terminal ubiquitin-binding domain and linking the cargoes with the autophagosome [67]. p62 and its cargoes are degraded following autolysosome formation [67, 68]. Therefore, it is generally accepted that increased levels of p62 result in the blockage of autophagic flux. However, p62 expression is also elevated under the conditions of oxidative stress and toxic stimuli [68, 69]. It was reported that p62 levels are regulated not only by its degradation but also at the transcriptional level, which may explain its increase after LCA treatment [69, 70]. It remains to be studied in the future how p62 is regulated at the transcriptional level after the exposure to LCA.

SolB treatment alone decreased the level of PINK1 protein, and this decrease may be regulated at its transcriptional level since SolB decreased hepatic mRNA levels of *Pink1*. SolB pretreatment decreased the increase in PARKIN levels and the ratio of LC3-II/LC3-I induced by LCA. Hence, we speculate that the protective effect of SolB against LCA-induced liver injury is not associated with PARKIN-dependent mitophagy. It is worth noting that p62 activated NRF2, which revealed that p62 interacted with the NRF2-binding site in Keap1, and that p62 accumulation resulted in activation of NRF2 [71]. SolB markedly increased the nuclear translocation of NRF2 [34]. And p62-NRF2-p62 regulatory loop was also regulated in the regulation of mitophagy under neurodegenerative disease conditions [72].

Taken together, this study reveals a mechanistic understanding of how SolB exerts hepatoprotection against LCA-induced liver injury. SolB modulates the RIP3-MLKL pathway, and improves mitochondrial function. While SolB upregulates mitochondrial dynamics proteins, the specific mechanism driving DRP1-dependent fission requires further investigation.

In summary, this study shows that the hepatoprotective effect of SolB against LCA-induced cholestatic liver injury is associated with improved mitochondrial function, independent of any influence on LCA-induced mitochondrial fragmentation, thereby offering new insight into SolB’s mechanism of hepatoprotection.

## Supplementary Information

Supplementary material 1. 

## Data Availability

No datasets were generated or analysed during the current study.

## Abbreviations

ANIT: Alpha-naphthylisothiocyanate
ALT: Alanine aminotransferase
AST: Aspartate aminotransferase
ALP: Alkaline phosphatase
DRP1: Dynamin-related protein1
ECL: Electrochemiluminescence
EM: Electron microscopy
FGF21: Fibroblast Growth Factor 21
FIS1: Fission1
FUNDC1: FUN14 domain-containing protein 1
GDF15: Growth differentiation factor 15
GCLC: Glutamate-cysteine ligase catalytic subunit
GCLM: Glutamate-cysteine ligase modifier subunit
GCDC: Glycochenodeoxycholate
HO-1: Heme Oxygenase 1
LCA: Lithocholic acid
LC3: Microtubule-associated protein light chain 3
MLKL: Mixed-lineage kinase domain-like protein
MFN: Mitofusin
MFF: Mitochondrial fission factor
MTCO1: Mitochondrially encoded cytochrome c oxidase subunit 1
NRF2: Nuclear factor erythroid 2-related factor 2
OA: Oleanolic acid
NQO1: NAD(P)H quinone oxidoreductase 1
OPA1: Optic atrophy 1
PXR: Pregnane X receptor
PGC-1α: Peroxisome proliferator-activated receptor γ co-activator 1α
PBC: Primary biliary cholangitis
PSC: Primary sclerosing cholangitis
PINK1: Phosphatase and tensin homolog (PTEN)-induced kinase 1
PARKIN: Parkin RBR E3 ubiquitin-protein ligase
RT-qPCR: Real-time quantitative PCR analysis
RIP1: Receptor-interacting protein1
RIP3: Receptor-interacting protein3
SOD: Superoxide dismutase
SolB: Schisandrol B
TBA: Total bile acid
TBILI: Total bilirubin
