# Supplementary material for: Schisandrol B protects against lithocholic acid-induced cholestatic liver injury in mice through mitochondrial biogenesis
Source: Chin Med. 2026 Apr 14;21:116. doi: 10.1186/s13020-026-01342-y (PMC13078053; doi:10.1186/s13020-026-01342-y)
Supplement: Supplementary file 1 — Supplementary material 1. [file 13020_2026_1342_MOESM1_ESM.docx]

**Schisandrol B protects against lithocholic acid-induced** **cholestatic liver injury in mice through mitochondrial biogenesis**

Xiao Yang^1#^, Hangfei Liang^2#^, Xuan Li^1^, Jianing Tian^2^, Shicheng Fan^1^, Min Huang^2^, Jianbo Wan^3^, Zhong Zuo^4^ Haibiao Guo^5*^, Huichang Bi^1*^

^1^NMPA Key Laboratory for Research and Evaluation of Drug Metabolism & Guangdong Provincial Key Laboratory of New Drug Screening & Guangdong-Hongkong-Macao Joint Laboratory for New Drug Screening, School of Pharmaceutical Sciences, Southern Medical University, Guangzhou 510515, China.

^2^Guangdong Provincial Key Laboratory of New Drug Design and Evaluation, School of Pharmaceutical Sciences, Sun Yat-sen University, Guangzhou 510006, China.

^3^State Key Laboratory of Mechanism and Quality of Chinese Medicine, Institute of Chinese Medical Sciences, University of Macau, Macao, China.

^4^School of Pharmacy, Faculty of Medicine, The Chinese University of Hong Kong, Hong Kong, Special Administrative Region, China.

^5^Hutchison Whampoa Guangzhou Baiyunshan Chinese Medicine Co., Ltd., Guangzhou, Guangdong 510515, China.

^#^: These authors contributed equally to this work and share the first authorship.

^*^**Correspondence：**

^*^Huichang Bi

School of Pharmaceutical Sciences, Southern Medical University, 1023# Shatai South Road, Baiyun District, Guangzhou 510515, P.R. China.

Email: [bihchang@smu.edu.cn](mailto:bihchang@smu.edu.cn)

^*^Haibiao Guo

Hutchison Whampoa Guangzhou Baiyunshan Chinese Medicine Co., Ltd., Guangzhou, Guangdong 510515, China.

Email: taylorghb@qq.com


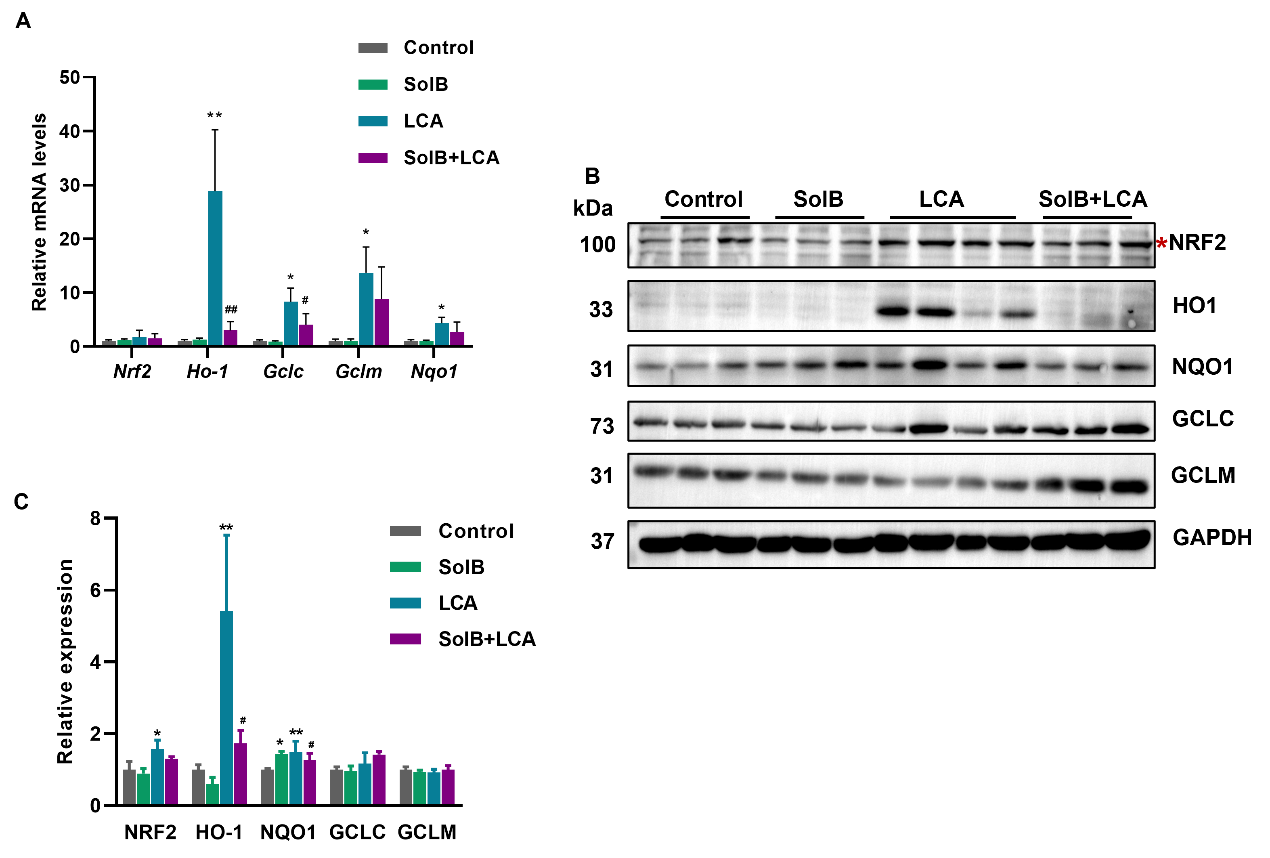


**Supplementary Fig.1** Effect of SolB on the levels of oxidative stress-related genes. **A** The mRNA expression of *Nrf2*, *Nqo1*, *Ho-1*, *Gclm* and *Gclc* were analyzed by qPCR. **B** The expression levels of NRF2, NQO1, HO-1, GCLM and GCLC in livers were determined by Western blot analysis. **C** Densitometry analysis of (B). The data are presented as mean ± SD (n=3-4). ^*^*P*< 0.05, ^**^*P*< 0.01 *versu*s the control group; ^#^*P*< 0.05, ^##^*P*< 0.01 *versus* the LCA group.
